# Firecracker Injuries During Chaharshanbeh Soori Festival In Iran: a Case Series Study

**DOI:** 10.5812/atr.9250

**Published:** 2013-06-01

**Authors:** Hamid Reza Hatamabadi, Ali Tabatabaey, Kamran Heidari, Mohamad Karim Khoramian

**Affiliations:** 1 Safety Promotion and Injury Prevention Research Center, Shahid Beheshti University of Medical Sciences, Tehran, IR Iran; 2 Emergency Department, Shahid Beheshti University of Medical Sciences, Tehran, IR Iran

**Keywords:** Wounds and Injuries, Emergency Medicine, Prevention and Control

## Abstract

On the last Wednesday of every year Iranians celebrate the sanctity of fire in the annual festival of Chaharshanbeh Soori. Each year many cases of firecracker-related injuries (FRI) are reported during this festival. The aim of this study was to assess the pattern of injuries and the frequency of disabilities during this period.

In 2011, a cross-sectional study was conducted at the emergency departments (EDs) of three educational hospitals in Tehran, to assess the extent and demographics of FRI. The age and sex of the patient, type of referral to the hospital, type of injury, its region and treatment process were recorded for each patient by the physicians. Data were analyzed by SPSS version 20. Thirty-five patients suffering from FRI were admitted to the hospitals during the festival. The majority of patients were under 30 years-old and most of them were male (83% male and 17% female). The injuries were mostly lacerations and cuts (n = 17, 49%) and scratches (n = 12, 34%). One patient suffered amputation. The most common site of the injuries were the hands (n = 13, 37%) followed by the face (n = 10, 29%). There were 10 patients (29%) with more than one site of injury. Twenty-one patients were hospitalized, 12 patients (34%) received outpatient treatment and two patients were referred to other hospitals. There are still many victims during Chaharshanbeh Soori festival despite efforts and legislations by the government. Education and raise of awareness among people especially for youth are the most important ways to prevent and reduce Red Wednesday injuries.

## 1. Introduction

One of the annual Iranian traditions is the celebration of Chaharshanbeh Soori. Held on the final Wednesday of the year, Chaharshanbe Soori or Red Wednesday is among the celebrations leading up to the Persian New Year or Nowruz on the spring Equinox. The word Chaharshanbeh means Wednesday and the word Soori means Red. The day is marked by different festivities and ceremonies celebrating the sanctity of fire, wishing away disease and disasters and wishing for health and good luck for the upcoming year. This ceremony dates back to many centuries ago (1). The fire in the perspective of Iranians is a symbol of something clear, clean, refreshing, and healthy. At Chaharshanbeh Soori people make a fire and jump over it while chanting a ritual song. Since ancient times, in addition to kindling fire, the celebration includes singing songs, dressing up and enjoying sweets and nuts. Yet unfortunately, in recent years the ceremony of Red Wednesday has been the source of many accidents and mishaps. What has gained the attention of citizens especially the youths and teenagers in recent years, is creating noise and frightening sounds through the explosion of flammable materials which are not less dangerous and destructive than explosive materials (2, 3). This issue has turned this tradition into a source of unwanted events and unpleasant accidents which threaten lives and properties. Firecracker-related injuries during the festival have been reported in the past (4, 5). This study has been conducted to report on the patient load induced by the festival on general hospitals in Tehran, rather than specific burns or eye injuries. Unlike most of the previous reports, this study is set in three general hospitals and focuses on the injuries reported on the day of the festival itself. This research can be an effective step in reducing the incidence of injuries of Red Wednesday.

## 2. Case Report

This cross-sectional study was conducted in the Emergency Departments (EDs) of Imam Hossein, Shohada Haftom Tir and Shohada Tajrish hospitals in Tehran during the 2011 Charshanbeh Soori day and night. The age, sex, type of referral to the hospital (call to emergency medical services (EMS), from other hospitals, outpatient), type of injury (Abrasion, scratch, amputation, burn, breakage, laceration and cut), sites of injury (ear, eye, face, leg, hand, head, neck, body), type of treatment (outpatient, hospitalization, referal to another hospital) were recorded for each patient with FRI by the physicians. SPSS version 20 was used to analyze the data and to perform statistical analysis where relevant. A P < 0.05 was considered statistically significant. Discrete variables were expressed as percentages. Continuous variables were reported as means and standard deviations. There were 35 firecracker-injured patients admitted during Charshanbeh Soori day and night. The mean age of patients was 24 years old (SD 12), ranging from 3 to 72 years old. Most patients were below 30 years old and were predominantly male (83% men, 17% women). Sixteen patients had been brought to the hospital by emergency medical services (call to 115) and 15 patients (43%) were admitted as outpatients. The information regarding type and site of injury is summarized in Table 1. There were 10 patients (29%) with more than one injury site. Twenty-one patients required hospitalization, 12 patients (34%) were treated as outpatients, and two patients were referred to other hospitals for further diagnostic and treatment services.

**Table 1.** Distribution of Injury Sites and Types

Demographic Features	No. (%)
**Gender**	
Male	29 (83)
Female	6 (17)
**Type of admission**	
Emergency Medical Services	16 (51)
Outpatient	15 (49)
**Type of injury**	
Lacerations	17 (49)
Scratches	12 (34)
Burns	5 (14)
Abrasions	5 (14)
Fractures	4 (11)
Amputations	1 (3)
**Injury site**	
Hands	13 (37)
Face	10 (29)
Eyes	7 (20)
Torso	5 (14)
Head	4 (11)
Ears	2 (6)

## 3. Discussion

Numerous publications have reported on the misuse of firecrackers as the main cause of injuries in various ceremonies held all over the world (6-10). Firecrackers are largely used among people during festivals for their sound, spark, shining and sudden explosion while lighting up the celebration mood. They are used during numerous festivals all over the world such as Tihar in Nepal, Ashura day in Morocco, Hari Raya in Malaysia, Day of Independence and Halloween in the USA, Bastille Day in France, Spanish Fallas and New Year's Day in Guatemala and China, Guy Fawkes Night in the UK and many other festivals in the world (7, 8, 11). Iran is a large country with a population of 70 million people. Despite a lot of government efforts there are many victims during Charshanbeh Soori festival every year (2). In the years 1980 to 1989 approximately 10000 individuals from the United States suffered injuries resulting from firecrackers; while the United States of America during the years 1990 to 2003 alone had 85800 injuries resulting from firecrackers (12). In the UK, the highest number of injuries were reported during Halloween (13). A total of 4447 patients with firecracker-related injuries received medical attention during the two days around the New Year celebrations at Denmark Hospitals (14). Greece has not been safe against firecracker- related injuries either. Annually from every 1000 child, 7 face these injuries, 70% of which occur in the age group of 10 to 14 years old (15). So, we can conclude that this type of injury affects both developed countries and developing countries. Imam Hussein and Shohada Haftom Tir Hospital are trauma center hospitals in Tehran and on the Red Wednesday of 2011 they admitted 35 firecracker-related injuries. Most of these patients were under 30 years old which indicates that firecrackers are more popular among children, adolescents and youth. This is confirmed by the 2009 Red Wednesday festival where more than 38 percent of the injured patients were students (16). In the USA, there is also a high level of reported firecracker-related injuries in children. In another study that was conducted on children under 15 years old, 0 to 50 % of the victims were from this population (17, 18). The studies which have been conducted in India also exhibit a high level of firecracker-related injuries among students (19). Only one study about FRI in Iran has looked at the entire range of injuries caused by firecrackers, in which the majority of injured patients were between 15-20 years old (3). Without a doubt, firecracker-related injuries are preventable, as we’ve observed in many countries over the past two decades where rules and restrictions for the use of flammable material have been applied. For example, some restrictions were applied in the UK which banned the use of such materials for people under 18 years old and it led to an 83 % reduction of injuries compared to the previous year (2004) (20). In New Zealand in 1992, the law imposed penalties for the sale and use of explosives (21). It has been reported that education alone does not protect against firework-related injuries and such legislative restraints are required (22). Despite restrictions on explosives and banning of firecrackers, still many injuries exist as an outcome of the use of these explosives. Northern Ireland hasn't used any legislation for the sale and use of firecrackers as compared to the UK and this freedom hasn’t resulted in a significant increase in firecracker injuries in this country (23). According to Iran’s Ministry of Health, 10 percent of people who suffer from eye injuries from Red Wednesdays require surgery (2, 4). Eye injuries, present in 27 percent of injured cases are the second problem that threatens people’s health in these events (9). In the present study, 20% of injuries were related to the eyes. The treatment of a patient suffering an eye injury will cost more than $50,000 during his lifetime (22). With that in mind, in the case of Red Wednesday for the final Wednesday of 2010, more than 130 people who suffered eye injuries referred to the Eye Specialist Hospital, Tehran University from which thirty people were at risk of blindness. According to the statistics of Iran’s Ministry of Health, in 2009, 1928 patients injured from Red Wednesday referred to hospitals whereas 1817 cases were referred in 2008; resulting in a 6% increase (16). In 2009, about 1601 cases of emergency admissions for hospitals related to the events of Red Wednesday from which about 487 individuals received treatments on site, and 114 individuals were dispatched to other hospitals; from this figure 1232 people were male and 369 people were female (16). According to the Department of Disaster Prevention for the Ministry of Health, the age group of 15 to 19 years old, with 21.9 % and the age group of 20 to 24 years old with 19.5 % in total contributed to 41 % of the injury events related to 2009. Also, the above mentioned injuries were 4.5% female (16). Similarly, in our study most of the injured patients (83%) were male. This is probably because male are more prone to take risks and have a higher presence on the streets. About 54% were injured during handling of the explosives, 19.2% while crossing the pedestrian, 8.7 % while watching, 5.9 % while jumping over fire, 1.7 % while on duty and 1.6 % while providing and producing the above-mentioned materials; 44.5 % of the injured patients had injury on their face and 40 % suffered from injuries on the rest of their body, 4 cases led to death in the emergency centers and 16 cases led to disabilities. In this study where the main types of injuries were lacerations, cuts and scratches and the most frequently injured body parts were hand (39.8%), face (21.1%) and eye (10.5%) which indicated that extremities are the main injured sites by firecrackers. Hands were the main sites of injury in previous studies in Saudi Arabia, Ireland, Denmark, Australia and England (24-28). There are several studies which show that the eyes are the main body part injured by fireworks. They also reported some amputations and blindness among injured patients (15, 29), yet there wasn't any blindness case in the present study and there was only one amputation case. The final Wednesday of the year has been celebrated by Iranians for thousands of years and has become a common tradition. Although it expresses the rich culture of Iranians, but the fire of this final Wednesday will always create problems for people using them and innocent bystanders alike. Although a lot is done by the government to prevent and reduce the occurrence of such damage yet there are still many victims who suffer. Education and raise of awareness for people particularly for the vulnerable groups is the most important way to prevent the events of Red Wednesday. The mass media, especially television, radio and newspapers can play an important role in the education of preventive actions to prevent and reduce such incidents. It seems that an effective way to avoid injuries of Red Wednesday is banning the delivery of explosive materials to the consumers.
